# Correlation between dental fluorosis risk and bone-specific alkaline phosphatase, osteocalcin, matrix metalloproteinase and parathyroid hormone in children

**DOI:** 10.5937/jomb0-48581

**Published:** 2024-11-16

**Authors:** Zheng Cao, Yuejian Ou, Yufeng Wang, Yuqing Zheng

**Affiliations:** 1 Affiliated Central Hospital of Huzhou University, Huzhou Central Hospital, Department of Stomatology, Huzhou, Zhejiang, China; 2 Huzhou University, School of Engineering, Huzhou, Zhejiang, China

**Keywords:** dental fluorosis, bone-specific alkaline phosphatase, osteocalcin, matrix metalloproteinase, parathyroid hormone, fluoroza zuba, alkalna fosfataza specifična za kosti, osteokalcin, matriks metaloproteinaza, paratiroidni hormon

## Abstract

**Background:**

The aim of this study was to investigate the association between dental fluorosis occurrence in children and bone metabolism-related indicators, including bone-specific alkaline phosphatase (BALP), osteocalcin (OC), matrix metalloproteinase (MMP-2, MMP-9, MMP-20), and parathyroid hormone (PTH).

**Methods:**

A total of 189 cases of school-age children who underwent health examinations in our hospital were enrolled, according to the presence or absence of dental fluorosis. They were divided into the fluorosis group (n=97) and fluoride-free group (n=92), and the serum BALP, OC, MMP-2, MMP-9, MMP-20, and PTH levels of the two groups were compared and relevant clinical data were collected. This study employed multivariate logistic regression to examine the factors associated with the development of dental fluorosis in children.

**Results:**

The urine fluoride levels, BALP, MMP-2, and MMP9 of the children in the fluorosis group were higher than fluoride-free group, and the mother's educational level, per capita annual household income, OC, and PTH were lower than fluoride-free group (P<0.05). Based on Spearman correlation analysis, a positive correlation was identified between the urinary fluoride level, the extent of dental fluorosis, and indicators such as BALP, MMP-2, and MMP-9. (r=0.618, 0.558, 0.567, 0.597, 0.602, 0.571, P<0.001), and negatively correlated with OC and PTH (r=-0.580, -0.603, -0.549, -0.515, P<0.001). As the urinary fluoride level and the extent of dental fluorosis increased, there was a gradual elevation in serum BALP, MMP-2, and MMP-9 levels in children, while OC and PTH levels gradually decreased (P<0.05). After adjusting for confounding factors, including urinary fluoride, maternal education level, and per capita annual household income, multivariate Logistic regression analysis showed that BALP, OC, MMP-2, MMP-9, and PTH were independently associated with the risk of dental fluorosis (P<0.05).

**Conclusions:**

High BALP, MMP-2, MMP-9, low OC, and PTH are independent factors affecting the occurrence of dental fluorosis and are related to the extent of dental fluorosis.

## Abstract (sr)

**Uvod:**

Cilj ove studije je bio da istraži povezanost između pojave dentalne fluoroze kod dece i indikatora vezanih za metabolizam kostiju, uključujući alkalnu fosfatazu specifičnu za kosti (BALP), osteokalcin (OC), matriks metaloproteinazu (MMP-2, MMP-9, MMP-20) i paratiroidni hormon (PTH).

**Metode:**

Ukupno je bilo 189 slučajeva dece školskog uzrasta koja su bila na zdravstvenim pregledima u našoj bolnici, a u smislu prisustva ili odsustva fluoroze zuba. Oni su podeljeni u grupu sa fluorozom (n=97) i grupu bez (n=92), a nivoi BALP, OC, MMP-2, MMP-9, MMP-20 i PTH u serumu dve grupe su bili upoređeni i prikupljeni su relevantni klinički podaci. Ova studija je koristila multivarijantnu logističku regresiju da bi ispitala faktore povezane sa razvojem dentalne fluoroze kod dece.

**Rezultati:**

Nivoi fluorida u urinu, BALP, MMP-2 i MMP-9 kode dece u grupi sa fluorozom bili su viši od grupe bez fluoroze, a nivo obrazovanja majke, godišnji prihod domaćinstva po glavi stanovnika, OC i PTH su bili niži nego u grupi bez fluoroze (P<0,05). Na osnovu Spirmanove korelacione analize, identifikovana je pozitivna korelacija između nivoa fluorida u urinu, stepena dentalne fluoroze i indikatora kao što su BALP, MMP-2 i MMP-9. (r=0,618, 0,558, 0,567, 0,597, 0,602, 0,571, P<0,001), i negativno korelira sa OC i PTH (r=-0,580, -0,603, -0,549, -0,515, P<0,001). Sa povećanjem nivoa fluorida u urinu i stepena dentalne fluoroze, došlo je do postepenog povećanja nivoa BALP, MMP-2 i MMP-9 u serumu kod dece, dok su nivoi OC i PTH postepeno opadali (P<0,05). Nakon prilagođavanja remetilačkih faktora, uključujući fluorid u urinu, nivo obrazovanja majke i godišnji prihod domaćinstva po glavi stanovnika, multivarijantna logistička regresiona analiza je pokazala da su BALP, OC, MMP-2, MMP-9 i PTH nezavisno povezani sa rizikom od dentalne fluoroze (P<0,05).

**Zaključak:**

Visok BALP, MMP-2, MMP-9, nizak OC i PTH su nezavisni faktori koji utiču na pojavu dentalne fluoroze i povezani su sa stepenom dentalne fluoroze.

## Introduction

Fluorine is a widely occurring element in nature, usually in the form of compounds, while the human digestive tract absorbs fluoride and accumulates mainly in bones or teeth [1]. Appropriate fluoride intake can help prevent dental caries, but excessive fluoride ingestion can lead to the development of dental fluorosis, especially in children’s permanent tooth eruption and mineralization stage [2]. Dental fluorosis is mainly manifested as spots or plaques on the surface of teeth caused by enamel coloring and damage, which not only affect the aesthetics but also have a greater impact on the chewing and food digestion function of the teeth, endangering children’s health [3]. Bone-specific alkaline phosphatase (BALP) is a stable indicator of osteoblast activity; Osteocalcin (OC) is involved in dentin formation and alveolar bone remodeling and has the highest contribution to the risk of dental fluorosis; Parathyroid hormone (PTH) regulates blood calcium concentration, participates in tooth development, and may be affected by fluoride exposure; Matrix metalloproteinase-2 (MMP-2) and MMP-9 play a key role in extracellular matrix degradation, promoting bone formation and participating in bone resorption and reconstruction, respectively. These biomarkers jointly affect bone and tooth health. Research findings indicate that tooth eruption and mineralization are closely linked to bone metabolism. Moreover, excessive fluoride intake has been found to induce alterations in bone metabolism-related indicators [4]. The relationship between dental fluorosis occurrence and bone metabolism remains incompletely understood. In this study, we focus on children diagnosed with dental fluorosis in our hospital to investigate the association between dental fluorosis occurrence and bone metabolism indicators. The findings aim to provide insights for preventing and treating dental fluorosis in clinical practice.

## Materials and methods

### General information

One hundred and eighty-nine cases of school-age children who underwent health examinations in our hospital from November 2021 to April 2023 were collected and included in the criteria: (1) Aged 7 to 12 years old; (2) For permanent families in the region; (3) Informed consent of the child and their parents. Exclusion Criteria: (1) There are bone metabolism or other diseases that affect calcium and phosphorus metabolism; (2) Those who have recently taken calcium or other drugs that affect bone metabolism; (3) There is a family history of high incidence of tumors; (4) Those who have received fluoride treatment in the past 1 year. Stomatologists evaluate the disease of dental fluorosis in children according to the Dean method [5] and divide them into fluorosis groups (n=97) and fluorosis-free groups (n=92), and one of them can be diagnosed with chalky, enamel staining or defects in the teeth [6].

### Methods

The basic information of children is collected, including gender, age, BMI, family situation (per capita annual income of the family, mother’s education level), and other information. Participants’ fasting morning urine samples were collected and stored at -4°C for analysis. The urine fluoride content was measured using the fluoride ion selective electrode method. The subject was drawn with fasting venous blood, centrifuged at 3000 r/min for 10 min to take the supernatant, and stored at -20°C for later use. The serum calcium, phosphorus, bone-specific alkaline phosphatase (BALP), osteocalcin (OC), and parathyroid hormone (PTH) levels were detected by the automatic biochemical instrument. Serum calcium was detected using the ortho-cresolphthalein complexone colorimetric method: the serum sample to be tested was mixed with a staining agent to produce a reaction, and the absorbance of the mixture was measured using a spectrophotometer at a wavelength of 520 nm. The concentration of calcium ions in the serum was obtained by comparing it with a standard curve. Serum phosphorus was detected using the phospho-molybdic acid UV method: the serum sample to be tested was mixed with a staining agent to produce a reaction, and the absorbance of the mixture was measured using a spectrophotometer at a wavelength of 650 nm. The concentration of phosphorus ions in the serum was compared with a standard curve. BALP Cai Yonghong chemiluminescence method was used to detect BALP in serum: an appropriate amount of BALP-specific antibody was added to the serum sample, allowing the antibody to bind to BALP in the serum to form an antigen-antibody complex. A luminous substrate was added, and the light signal generated during the reaction was detected using a chemiluminescence analyzer. The concentration of BALP in serum was obtained by comparing it with a standard curve. OC and PTH were detected using radioimmunoassay: an appropriate amount of serum sample and radioactively labeled antigen or antibody were mixed, and unlabeled antigen or antibody was added to compete with the target molecule in the sample for binding. The radioactive intensity was detected, and the concentration of OC or PTH in serum was calculated by comparing it with a preset standard curve. MMP-2 and MMP-9 were detected using ELISA: the prepared serum was added to the wells of an ELISA plate containing MMP-2 or MMP-9 antibodies, allowing specific binding to occur. Then, enzyme-labeled secondary antibodies were added, and after sufficient reaction, each well’s optical density (OD) was measured using an enzyme microplate reader. The concentration of MMP-2 and MMP-9 was calculated based on the OD value and concentration of standard samples. The reagent kit was purchased from RD Corporation in the United States.

According to the actual measurement range of urine fluoride in children with fluoride disease with fluorosis, 0.62–2.94 mg/L, four dose groups were set up with an equal ratio spacing of 1.5 times, namely <1.2 mg/L, 1.2 mg/L~, 1.8 mg/L~, 2.4 mg/L~. The diagnostic criteria for dental fluorosis, as outlined in the clinical guidelines WST208-2011, were used for clinical assessment; children with dental fluorosis were divided into three groups of mild, moderate and severe (mild, chalky opaque part <1/4 of the affected teeth; Moderate, chalky opaque parts throughout the affected tooth; Severe, severe enamel involvement, the fusion of defects, and dental morphology are also affected), and differences in bone metabolism in children with different levels of urine fluoride and severity of fluorosis were analyzed.

### Statistical methods

SPSS 22.0 software package analyzes data, conforms to normal distribution measurement data in (x̄±s) representation, intergroup comparison line t-test; The counting data is expressed as a percentage of »%,« and the intergroup comparison line 2 test; Rank sum tests are used for data that do not satisfy the normal distribution; The correlation analysis was tested by Spearman correlation, and the multivariate analysis affecting the occurrence of dental fluorosis was tested by Logistic regression. Note P< 0.05 for the difference is statistically significant.

### Study results

### Two groups of general demographic characteristics

The urine fluoride levels, BALP, MMP-2, and MMP-9 of the children in the fluorosis group were higher than those in the fluorosis-free group, and the mother’s educational level, per capita annual household income, OC, and PTH were lower than those fluoride- free group (P<0.05), and there was no obvious difference in sex, age, and BMI between 2 groups (P>0.05) as shown in Table 1.

**Table 1:** General demographic characteristics of 2 groups [n (%), M(P25, P75)]. Note: BMI, body mass index; BALP, bone-specific alkaline phosphatase; OC, osteocalcin; MMP-2, matrix metalloproteinase-2; MMP-9, matrix metalloproteinase-9; PTH, parathyroid hormone

Factor	Dental fluorosis group (n=97)	Fluorosis-free group (n=92)	χ^2^/t/Z	P
Gender				
male	47 (48.45)	54 (58.70)	1.991	0.158
female	50 (51.55)	38 (41.30)		
Age (years)	9.94±1.26	9.85±1.20	0.502	0.616
BMI (kg/m^2^)	17.14±2.05	17.62±1.93	1.655	0.100
Mother’s education level				
Junior high school and below	42 (43.30)	18 (19.57)	15.937	0.000
Vocational high school, high school	31 (31.96)	29 (31.52)		
College degree or above	24 (24.74)	45 (48.91)		
Annual household income per				
< 10,000 yuan	49 (50.52)	22 (23.91)	14.910	0.001
10,000 to 50,000 yuan	35 (36.08)	46 (50.00)		
> 50,000 yuan	13 (13.40)	24 (26.09)		
Urinary fluoride (mg/L)	1.73 (0.79, 2.39)	1.22 (0.37, 2.08)	3.405	0.007
BALP (μg/L)	113.59±16.76	98.94±25.36	4.708	0.000
OC (ng/mL)	11.56±3.04	12.78±3.84	2.426	0.016
MMP-2 (ng/mL)	9.49±2.37	7.86±3.04	4.122	0.000
MMP-9 (ng/mL)	10.43±3.03	8.46±2.84	4.603	0.000
PTH (ng/dL)	13.98±3.27	14.96±3.23	2.078	0.039

### Comparison of bone metabolism indices in children with different urinary fluoride

**Figure 1:**
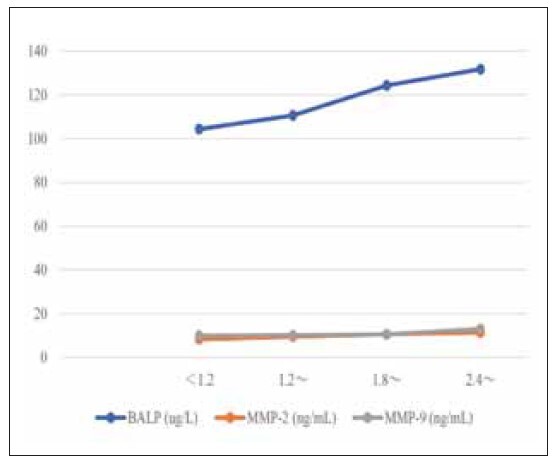
The line chart of the relationship between the severity of dental fluorosis and the changes of BALP, MMP-2, and MMP-9. Note: BALP, bone-specific alkaline phosphatase; MMP-2, matrix metalloproteinase-2; MMP-9, matrix metalloproteinase-9.

**Figure 2:**
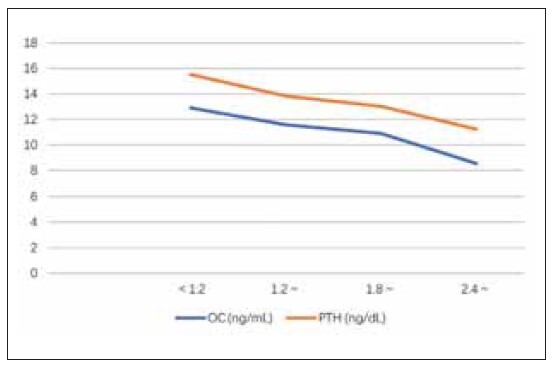
The line chart of the relationship between the severity of dental fluorosis and the changes in OC and PTH Note: OC, osteocalcin; MMP-2; PTH, parathyroid hormone.

With the increase in urine fluoride level, serum BALP, MMP-2, and MMP-9 gradually increased, and OC and PTH gradually decreased (P<0.05). Based on Spearman correlation analysis, a positive correlation was found between urinary fluoride levels and indicators such as BALP, MMP-2, and MMP-9 (r=0.618, 0.558, 0.567, P<0.001), and were negatively correlated with OC and PTH (r=-0.580, -0.603, P<0.001), as shown in Table 2, Figure 1 and Figure 2.

**Table 2:** Comparison of bone metabolism indices in children with different urinary fluoride levels. Note: BALP, bone-specific alkaline phosphatase; OC, osteocalcin; MMP-2, matrix metalloproteinase-2; MMP-9, matrix metalloproteinase-9; PTH, parathyroid hormone.

Urinalysis (mg/L)	n	BALP (μg/L)	OC (ng/mL)	MMP-2 (ng/mL)	MMP-9 (ng/mL)	PTH (ng/dL)
<1.2	36	104.36±12.61	12.90±3.26	8.32±2.24	9.86±2.72	15.52±3.24
1.2~	30	110.60±13.54	11.58±2.36	9.47±2.08	10.07±2.94	13.83±2.95
1.8~	19	124.32±16.52	10.90±2.17	10.56±2.04	10.48±3.09	13.02±2.74
2.4~	12	131.77±12.35	8.54±2.84	11.36±2.16	12.96±3.15	11.23±2.64
*F*	-	16.836	7.997	8.198	3.625	7.244
*P*	-	0.000	0.000	0.000	0.016	0.000

### Comparison of bone metabolism indicators in children with different severity of dental fluorosis

As the extent of dental fluorosis increased, there was a gradual elevation in serum levels of BALP, MMP-2, and MMP-9 in children, and the OC and PTH decreased (P<0.05). Spearman correlation analysis showed that the extent of dental fluorosis was positively correlated with BALP, MMP-2, MMP-9 (r=0.597, 0.602, 0.571, P<0.001), and was negatively correlated with OC and PTH (r=-0.549, -0.515, P<0.001), as shown in Table 3.

**Table 3:** Comparison of bone metabolism indices in children with different dental fluorosis severity. Note: BALP, bone-specific alkaline phosphatase; OC, osteocalcin; MMP-2, matrix metalloproteinase-2; MMP-9, matrix metalloproteinase-9; PTH, parathyroid hormone.

Dental fluorosis	n	BALP (μg/L)	OC (ng/mL)	MMP-2 (ng/mL)	MMP-9 (ng/mL)	PTH (ng/dL)
Mild	61	107.08±13.41	12.27±2.98	8.75±2.24	9.94±2.76	14.78±3.31
Moderate	21	122.15±17.69	11.29±2.48	10.43±2.05	10.30±3.11	13.28±2.55
Severe	15	128.10±13.69	9.04±2.72	11.21±2.07	12.60±3.25	11.66±2.76
*F*	-	17.398	7.899	10.199	5.045	6.859
*P*	-	0.000	0.001	0.000	0.008	0.002

### Multivariate logistic regression analysis affecting the occurrence of dental fluorosis

After correcting for the mixed factors such as urinary fluoride, maternal education level, and per capita annual household income, multivariate Logistic regression analysis showed that BALP, OC, MMP-2, MMP-9, and PTH were independently associated with the risk of dental fluorosis (P<0.05), as shown in Table 4.

**Table 4:** Multivariate logistic regression analysis affecting the occurrence of dental fluorosis. Note: BALP, bone-specific alkaline phosphatase; OC, osteocalcin; MMP-2, matrix metalloproteinase-2; MMP-9, matrix metalloproteinase-9; PTH, parathyroid hormone.

Factor	β	S.E.	Wald	*P*	*OR*	*95%CI*
BALP	0.854	0.269	10.079	0.001	2.349	1.386~3.980
OC	0.890	0.235	14.343	<0.001	2.435	1.536~3.860
MMP-2	1.111	0.276	16.204	<0.001	3.037	1.768~5.217
MMP-9	1.329	0.322	17.035	<0.001	3.778	2.009~7.100
PTH	0.747	0.250	8.928	0.003	2.111	1.293~3.445

## Discussion

Dental fluorosis is mainly manifested by plaque and defects on the enamel of the tooth, which not only causes dental damage but also damages the mental health of children due to aesthetic problems [7]. Dental fluorosis is a manifestation of bone phase injury in fluorosis characterized by high bone turnover, which includes abnormal osteoblasts and osteoclasts’ abnormal function [8]. Bone turnover imbalance is often reflected in the abnormality of bone metabolism index [9]; this study shows that the fluorosis group had higher BALP, MMP-2, and MMP-9 than the fluoride-free group and lower OC and PTH than the fluoride-free group, indicating that compared with children without fluorosis, there are obvious bone metabolic index abnormalities in children with fluorosis.

BALP is an alkaline phosphatase synthesized by liver and osteoblasts in the human body, whose metabolism is not affected by liver disease, and its expression reflects the activity of osteoblasts with certain stability and specificity [10]. At present, BALP is often used as an indicator of osteoblast activity in clinical practice, and it can also reflect the bone conversion rate to a certain extent [11]. OC has been confirmed to be involved in dentin formation and alveolar bone remodeling, and an analysis of the contribution of bone metabolism indexes to the risk of dental fluorosis shows that OC has the highest contribution to the occurrence of dental fluorosis, even exceeding blood calcium, blood phosphorus and other indicators [12]. PTH is a common indicator of regulating blood calcium concentration in the body and increases the concentration of calcium ions, and reduces phospholipid content [13]. In addition, PTH plays an important role in tooth eruption, mineralization and root resorption processes. Fluoride exposure has also been shown to interfere with PTH levels in rat serum [14]. As important members of the MMPs family, MMP-2 and MMP-9 play an important role in extracellular matrix physiology and pathological degradation, and related studies have shown that in addition to the abnormal function of osteoblasts and osteoclasts, there are also changes in the structure of the cell matrix [15]. MMP-2 has been reported to promote the migration and survival of osteoblasts, thereby participating in bone formation [16]. MMP-9, on the other hand, lowers the extracellular matrix and is involved in bone resorption and reconstruction [17]. Taking the level of urine fluoride as the internal loading index and the extent of dental fluorosis the clinical effect index, it was found that with the increase in the urinary fluoride level and the extent of dental fluorosis, the serum BALP, MMP-2 and MMP-9 of the children gradually increased, and the OC and PTH gradually decreased, suggesting that BALP, MMP-2, MMP-9, OC, and PTH were closely related to the occurrence and development of dental fluorosis. Some scholars believe that excess fluoride can contribute to the occurrence of dental fluorosis by influencing bone metabolic activity [18]. Spearman correlation analysis showed that the urinary fluoride level and the extent of dental fluorosis were positively correlated with BALP, MMP-2, and MMP-9, and negatively correlated with OC and PTH, suggesting that there was a clear dose-effect relationship between BALP, MMP-2, MMP-9, OC, PTH, and urine fluoride levels. Serum ALP, BALP, and OC levels have been shown to be related to the dose and timing of the body’s fluoride exposure [19], consistent with this study. After correcting the mixed factors such as urinary fluoride, maternal education level, and per capita annual income of the family, the multivariate Logistic regression analysis showed that BALP, OC, MMP-2, MMP-9, PTH were independently related to the risk of dental fluorosis, suggesting that BALP, OC, MMP-2, MMP-9, PTH were closely related to the occurrence of dental fluorosis. The monitoring of bone metabolism indicators in children in high-fluoride areas should be paid attention to in clinical work to prevent and treat dental fluorosis effectively.

In summary, high BALP, MMP-2, MMP-9, and low OC, PTH are independent factors affecting the occurrence of dental fluorosis and are related to the urinary fluoride level and the extent of dental fluorosis. However, there are still deficiencies in this study; some studies believe that after fluoride enters the human body, it can first stimulate osteoblasts to promote bone conversion, and with the prolonged duration of fluoride exposure, bone conversion slows down, serum bone metabolism index can appear a certain degree of correction [20], suggesting that the correlation between bone metabolic indicators and dental fluorosis needs to be dynamically observed for a long time, thus drawing further reliable conclusions.

## Dodatak

### Acknowledgments

This study was supported by the Project of Huzhou Science and Technology Bureau (2020GYB11).

### Conflict of interest statement

All the authors declare that they have no conflict of interest in this work.
